# Sustainable chemical science and engineering: celebrating the 130th anniversary of Tianjin University

**DOI:** 10.1039/d5sc90214b

**Published:** 2025-10-10

**Authors:** Jinlong Gong, Yingjin Yuan, Naiqin Zhao, Shi-Zhang Qiao

**Affiliations:** a School of Chemical Engineering & Technology, Key Laboratory for Green Chemical Technology of Ministry of Education, Tianjin University, Collaborative Innovation Center for Chemical Science & Engineering (Tianjin) Tianjin 300350 China; b International Joint Laboratory of Low-carbon Chemical Engineering of Ministry of Education Tianjin 300350 China; c State Key Laboratory of Synthetic Biology, Frontiers Science Center for Synthetic Biology (Ministry of Education), School of Synthetic Biology and Biomanufacturing, Tianjin University Tianjin, 300072 China yjyuan@tju.edu.cn; d Tianjin Normal University Tianjin 300350 China jlgong@tju.edu.cn; e Tianjin Key Laboratory of Composite and Functional Materials, School of Materials Science and Engineering, Tianjin University Tianjin 300350 China; f State Key Laboratory of High Performance Roll Materials and Composite Forming, Tianjin University Tianjin 300350 China nqzhao@tju.edu.cn; g School of Chemical Engineering, The University of Adelaide Adelaide SA 5005 Australia s.qiao@adelaide.edu.au

## Abstract

Jinlong Gong, Yingjin Yuan, Naiqin Zhao and Shi-Zhang Qiao introduce the Royal Society of Chemistry cross-journal themed collection celebrating the 130th anniversary and a century of chemical engineering, at Tianjin University in 2025.
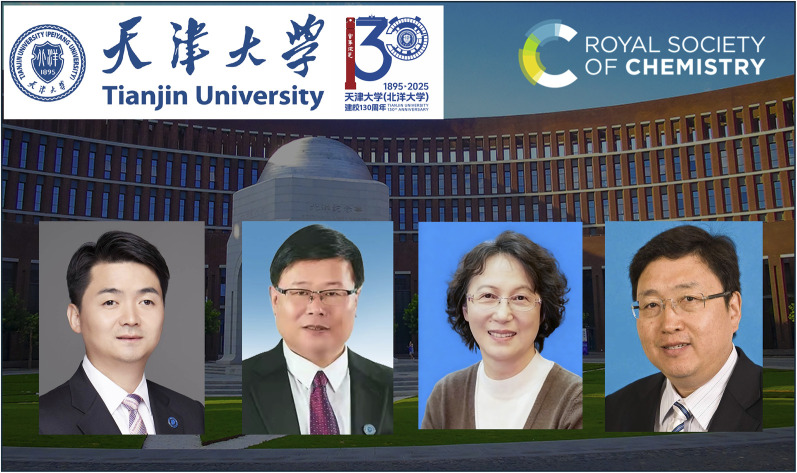

Founded in 1895 as Imperial Tientsin University, later known as Peiyang University, Tianjin University (TJU) was the first modern higher education institution in China. Over the past 130 years, it has evolved into a leading research university, guided by its motto, “Seeking Truth from Facts (
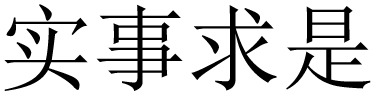
)” and cultivating generations of scholars who have advanced the frontiers of science and engineering. Renamed Tianjin University in 1951, it became one of China’s first 16 National Key Universities and a cornerstone of national initiatives such as the “211” and “985” projects. Today, TJU continues to drive scientific and technological innovation, particularly in chemical engineering and technology, producing leaders across academia, industry, and government.

Marking its 130th anniversary in 2025, TJU looks to the future of sustainable chemical science and engineering. The university has pioneered research across catalysis, energy conversion, advanced materials, and biomolecular design. To celebrate this milestone, the Royal Society of Chemistry presents a cross-journal themed collection, showcasing TJU’s interdisciplinary spirit and its commitment to innovative solutions for a more sustainable world. As of 20 September 2025, a total of 27 articles in this collection have been published online. Several other articles are still under review and will be added to the collection as they are published throughout 2025.

## Catalysis for sustainable chemical transformations

Catalysis is the cornerstone of modern chemical science, providing pathways to valorize waste, close carbon cycles, and drive energy transformations. Advances in catalyst design not only reduce environmental impact but also enable the integration of renewable resources into chemical production. Lei *et al.* demonstrate an atom-economical method for thioamide synthesis by valorizing hydrogen sulfide waste streams using N-doped carbon catalysts (*EES Catal.*, https://doi.org/10.1039/d5ey00110b). Wang *et al.* employ high-throughput DFT screening to identify bimetal-modified Ti-MOFs capable of selectively reducing CO_2_ to ethanol, pinpointing Ti(Nb)-ATA as a promising candidate (*J. Mater. Chem. A*, https://doi.org/10.1039/d5ta03415a). The role of microenvironments in catalysis is exemplified by Dong *et al.*, who show that hydrophobic SnS_2_ catalysts enhance CO_2_ reduction by regulating the local CO_2_/H_2_O ratio (*Green Chem.*, https://doi.org/10.1039/d5gc00635j). Similarly, Qin *et al.* demonstrate tailoring hydrophobicity of immobilized molecular catalysts, achieving high CO selectivity at industrial current densities (*Chem. Sci.*, https://doi.org/10.1039/d4sc08219b). Further pushing the boundaries, Zhao *et al.* designed an MOF-derived heterojunction with precisely positioned cocatalysts for the selective oxidation of methane to liquid fuels, achieving remarkable selectivity and activity (*Chem. Sci.*, https://doi.org/10.1039/d5sc04771d). Beyond molecular design, process intensification is key to green chemistry. Wang *et al.* demonstrate this by establishing a continuous-flow photochemical system for the solvent-free synthesis of quadricyclane from norbornadiene. This approach achieved near-unity conversion and yield with low photosensitizer loading, supported by a robust kinetic model and CFD simulations that guided the design of a scalable reactor with a high daily output (*React. Chem. Eng.*, https://doi.org/10.1039/d5re00188a). These studies underscore how fundamental insights into active sites and local environments as well as process intensification are driving the development of catalytic processes for a circular carbon economy.

## Solar and electrochemical energy technologies

Solar and electrochemical energy technologies are at the forefront of the transition toward a sustainable, low-carbon future. By combining advances in molecular engineering, interface design, and catalytic innovation, researchers are unlocking new possibilities for efficient energy harvesting, storage, and utilization. On the storage side, Jia *et al.* introduce oxime-based organic cathodes that resist dissolution and deliver remarkable cycling stability over 12 000 cycles (*J. Mater. Chem. A*, https://doi.org/10.1039/d5ta01767j). Kong *et al.* explore the architecture of bipolar all-solid-state batteries, where minimized inactive components and optimized interfaces pave the way to safer and more efficient pouch-cell designs (*EES Batteries*, https://doi.org/10.1039/d5eb00126a). Furthermore, the separation of hydrogen isotopes is critical for nuclear energy. Wang *et al.* provide a comprehensive review of electrochemical protium and deuterium (H/D) separation technologies, positioning them as a transformative alternative to traditional industrial methods (*J. Mater. Chem. A*, https://doi.org/10.1039/d5ta02337h). Molecular solar thermal (MOST) systems offer a complementary pathway for storing and releasing solar energy. Xu *et al.* provide a comprehensive review of MOST devices, from photochemical switches to wearable heating fabrics (*Energy Environ. Sci.*, https://doi.org/10.1039/d5ee02556g), and further enhance the concept by designing phase-change azobenzene derivatives with record energy densities of 525 kJ mol^−1^ (*Green Chem.*, https://doi.org/10.1039/d5gc03447g).

In parallel, solar energy conversion, particularly organic photovoltaics (OPVs), is undergoing rapid transformation. Gu *et al.* report a cathode interlayer based on an indandione-terminated quinoidal compound, achieving a champion efficiency of 19.05% in OPVs (*Mater. Horiz.*, https://doi.org/10.1039/d5mh00536a). Zhang *et al.* revisit poly(3-alkylthiophene)-based systems, highlighting their cost-effectiveness and environmental compatibility in next-generation OPVs and photodetectors (*Mater. Horiz.*, https://doi.org/10.1039/d5mh01115a). Complementing these advances, Zuo *et al.* review intrinsically stretchable OPVs, showcasing strategies that combine high efficiency with mechanical robustness (*Energy Environ. Sci.*, https://doi.org/10.1039/d5ee01504a). Gong *et al.* reveal how phenothiazine conformations govern the trade-off between voltage and stability in organic cathodes (*J. Mater. Chem. A*, https://doi.org/10.1039/d5ta02323h), while Zhao *et al.* demonstrate how ligand effects shift oxygen adsorption pathways in carbon catalysts, achieving remarkable ORR performance (*Energy Environ. Sci.*, https://doi.org/10.1039/d5ee01407g). Together, these contributions demonstrate how chemistry is driving progress across the solar-to-electricity spectrum and electrochemical storage technologies. From robust batteries and solar thermal fuels to flexible, high-efficiency photovoltaics, the integration of molecular design with device engineering is reshaping the future of sustainable energy.

## Rational design of advanced materials

The rational design of materials is increasingly accelerated by computation, machine learning, and deep mechanistic insight. These tools are not only guiding catalyst development but also unlocking new classes of functional materials. In the realm of energy materials, Yang *et al.* used molecular dynamics simulations to decode how the monomer structure of alkaline polymer electrolytes (APEs) influences the electrical double-layer at the electrode interface, revealing that rigid monomers form distinct, ordered structures that enhance catalytic effects (*Chem. Sci.*, https://doi.org/10.1039/d5sc00492f). Gu *et al.* present a multimodal machine learning framework that predicts the stability and synthesizability of bimetallic materials, validated experimentally (*Chem. Sci.*, https://doi.org/10.1039/d5sc04386g). Zhao *et al.* proposed a theoretical design strategy for 2D single-molecule magnetic materials using endohedral metallofullerenes, with DFT calculations revealing that fullerene confinement induces single-electron metal–metal bonding to enhance magnetic properties (*Chem. Sci.*, https://doi.org/10.1039/d5sc01607j).

Wang *et al.* show how the symmetry of polymer supports induces dipole effects that tune the activity of single-atom photocatalysts (*Chem. Sci.*, https://doi.org/10.1039/d5sc02256h). Li *et al.* introduce a sensing paradigm exploiting non-equilibrium hot electrons in heterostructures for ultrasensitive cancer biomarker detection (*Chem. Sci.*, https://doi.org/10.1039/d5sc05009j). Ma *et al.* identify electron–phonon coupling as a hidden determinant of phosphorescence efficiency, establishing a new design principle for organic room-temperature phosphorescent materials (*Chem. Sci.*, https://doi.org/10.1039/d5sc02149a). These examples demonstrate how advanced materials research, powered by computation and mechanistic understanding, provides tools for innovations across catalysis, energy, and electronics.

## Bio-inspired solutions for health and the environment

Drawing inspiration from nature provides a powerful route to designing multifunctional, efficient, and environmentally compatible materials and systems. The blending of biology with chemistry opens opportunities in catalysis, coatings, sensing, and therapeutics. Li *et al.* investigate polymer encapsulation of enzymes through simulations, revealing a hierarchical assembly mechanism for nanocapsules (*Chem. Sci.*, https://doi.org/10.1039/d5sc02655e). He *et al.* review advances in engineering globular proteins into platforms that combine biological functionality with tailored mechanical performance for applications in tissue engineering and soft electronics (*Mater. Horiz.*, https://doi.org/10.1039/d5mh01107h). In antifreeze-inspired materials, Guo *et al.* review AFP-mimetic coatings that offer durable anti-icing performance (*J. Mater. Chem. A*, https://doi.org/10.1039/d5ta03663a). Tao *et al.* develop an activatable probe for bacterial imaging and photodynamic inactivation, while Li *et al.* advance cancer photodynamic therapy with a nuclear-targeted photosensitizer effective even under hypoxia (*Chem. Sci.*, https://doi.org/10.1039/d5sc00858a; *Chem. Sci.*, https://doi.org/10.1039/d5sc02476e). These works exemplify how mimicking nature’s principles can lead to sustainable and life-saving technologies.

### Outlook

This collection highlights the recent advances in sustainable chemical science and engineering, encompassing catalysis, energy technologies, computational materials, and bio-inspired strategies. These studies demonstrate how chemistry addresses pressing environmental and societal challenges worldwide. As Tianjin University celebrates its 130th anniversary, it reaffirms its commitment to nurturing talent, fostering innovation, and promoting interdisciplinary and international collaboration. By embracing emerging tools such as artificial intelligence, high-throughput experimentation, and circular economy principles, the next century of chemical science promises sustainable, efficient, and transformative solutions for society and the planet.

The Guest Editors would like to thank all the authors for their excellent contributions and the referees for their dedication and responsibility. We also would be happy to acknowledge Dr May Copsey, Dr Guanqun Song and the other Editorial Board Members of the Royal Society of Chemistry journals for their support, cooperation and valuable advice. We are indebted to Dr Tingyu Huang for her assistance in preparing this issue throughout all the stages.

